# Evolving pathways towards water security in the Vietnamese Mekong Delta: An adaptive management perspective

**DOI:** 10.1007/s13280-024-02045-0

**Published:** 2024-06-29

**Authors:** Thong Anh Tran, Dung Duc Tran, Oc Van Vo, Van Huynh Thanh Pham, Hieu Van Tran, Ming Li Yong, Phu Viet Le, Phu Thanh Dang

**Affiliations:** 1 Fenner School of Environment and Society, College of Science, The Australian National University, Canberra, Australia; 2 Climate Change Institute, An Giang University, Vietnam National University Ho Chi Minh City (VNU-HCM), Long Xuyen City, An Giang Province Vietnam; 3 National Institute of Education, Earth Observatory of Singapore and Asian School of the Environment, Nanyang Technological University, Singapore, Singapore; 4 Centre of Water Management and Climate Change, Institute for Environment and Resources, Vietnam National University Ho Chi Minh City (VNU-HCM), Ho Chi Minh City, Vietnam; 5 Faculty of Agriculture and Natural Resources, An Giang University, Vietnam National University Ho Chi Minh City (VNU-HCM), Long Xuyen City, An Giang Province Vietnam; 6 Research Program, East-West Centre, Honolulu, HI USA; 7 Fulbright School of Public Policy and Management, Fulbright University Vietnam, Ho Chi Minh City, Vietnam

**Keywords:** Adaptive management, Climate change, Saltwater intrusion, Vietnamese Mekong Delta, Water scarcity, Water security

## Abstract

The interplay of climate change, upstream hydropower development, and local water engineering interventions for agricultural production contributes substantially to the transformation of waterscapes and water scarcity in the Vietnamese Mekong Delta. This paper aims to examine how these dynamics are linked to the paradigm shift in water management in An Giang and Ben Tre, the two ecologically distinct provinces that face serious water scarcity in the delta. We used the adaptive management concept to examine how state-led policy directions from food security towards water security enable change in water management that gives priority to water retention. While policy learning is evident, questions remain about how this ad-hoc solution could help address the presently acute water scarcity and water security over the long term. The paper advocates achieving water security should focus not only on diplomatic interventions into upstream climate-development complexities but also local water-livelihood politics.

**Supplementary Information:**

The online version contains supplementary material available at 10.1007/s13280-024-02045-0.

## Introduction

The Anthropocene era as a geological epoch named to reflect how “humans themselves are having a vast and significant effect on the Earth” (Cook et al. 2015, p. 2), of which one dimension is characterised by significant human interference in water systems, including climate forcing, water withdrawal, dam construction, and land use changes (Falkenmark et al. 2019; Nicholls et al. 2020). Due to negative externalities derived from these anthropogenic interventions, many countries, especially those in the Global South, have become hotspots for water scarcity (Veldkamp et al. 2017; Huggins et al. 2022; FAO and AWP 2023). In these areas, while water demands are constantly increasing, nearly 80 per cent of their populations are subject to escalating effects of water insecurity that threaten their livelihoods (Vörösmarty et al. 2010; Williams and Carrico 2017; Shah 2021).

The Vietnamese Mekong Delta (VMD) is currently confronting critical water challenges characterised by the complex interplay of climate change, upstream hydropower development, and the state-led water engineering interventions for intensive rice production (Tran et al. 2018; Tran and Tortajada 2022; Smajgl et al. 2023). Disruptions to the Mekong River’s hydrological flows in the floodplains and saltwater intrusion in the coastal zones have become more frequent, resulting in a profound transformation of the delta’s waterscapes (Eyler 2019; Park et al. 2022; Tran et al. 2022a). Water scarcity is specifically perceived as the most prominent issue, placing disproportionately high pressure on agrarian societies (Tran and Cook 2024). While these challenges are increasingly obvious, there remains a lack of empirical understanding of how the evolving processes of water infrastructure development are linked to the adaptive management approach that has been put in place in the VMD.

Water challenges make a call for the adoption of adaptive water management approaches, which emphasise the need for iterative learning, policymaking, and implementation. At the global scale, a paradigm shift in water management has increasingly attracted scholarly and policy attention as to deploy proper water management solutions to address emerging water complexities (Schoeman et al. 2014; Akhmadiyeva and Abdullaev 2019; Bjornlund et al. 2021). The history of agricultural development in the VMD has witnessed the unceasing ‘taming’ processes of its water regimes dominated by the adoption of technical interventions—from opening-up (building canals) and closing-off (building dykes), to sealing off (building sluices) of the delta (Miller 2007; Tran et al. 2022b) to deal with floods and saltwater intrusion (Tran 2020; Tran et al. 2021). While most studies focus on how these processes involved prescriptive actions to resolve water problems at a specific point in time (Nguyen et al. 2017; Park et al. 2020), little is known about change in decision-making and learning in water management in attempts to curb water scarcity and achieve water security in the delta.

This study fills this gap by applying Pahl-Wostl’s conceptualisation of adaptive management to examine how water management is embedded in the surging climate and development complexities in the VMD, a new frontier of water-related risk and adaptation management in the Mekong region. Here, adaptive management is defined as “a systematic process for improving management policies and practices by learning from the outcomes of implemented management strategies” (Pahl-Wostl 2008, p. 1). Based on a qualitative study undertaken in two provinces (An Giang and Ben Tre) in the VMD, we demonstrate that the state’s investment in large-scale water infrastructure (e.g. dykes and canals) extending from the floodplains to the coastal zones in support of food security and rice-exporting demands makes substantial contribution to the transformation of waterscapes as well as water scarcity, leading to the paradigm shift in water management. From the perspective of adaptive management, the paper contributes to advancing the theoretical and empirical understandings of how the Mekong’s ‘fluid geographies’ under the detrimental effects of climate change and hydropower development shape human–water relations, and ways rural societies respond to the delta’s transforming waterscapes. It calls for policy needs to address the emerging water challenges, while acknowledging the pivotal role of adaptive management in leveraging efforts towards achieving water security in the long term.

## Conceptual framework

### Water security

Water security, since being first introduced in the early 2000s, has gained prominence in international and national water policy arenas (Clement 2013). The concept, as defined by Grey and Sadoff (2007, p. 545), is “the availability of an acceptable quantity and quality of water for health, livelihoods, ecosystems and production, coupled with an acceptable level of water-related risks to people, environments and economies.” Water security is also conceptually concerned with the capacity and knowledge of a population to gain access to and utilise water (van Noordwijk et al. 2016; Grafton 2017). Put in the broader context of the 2030 Agenda for Sustainable Development (UN DESA 2015), water security speaks directly to Goal 2 which aims to end hunger, achieve food security and improved nutrition, and promote sustainable agriculture and Goal 6 to ensure availability and sustainable management of water and sanitation for all (Sadoff et al. 2020; Taka et al. 2021).

However, there have been critical debates on how water security is defined. The concept embraces different framings, with the initial focus the quantity, availability, and quality of water, human health, and ecological concerns (Cook and Bakker 2012). In the framing of sustainability, water security is defined as access to sufficient safe water that allows a clean, healthy, and productive life, while ensuring the protection and enhancement of natural environments (Global Water Partnership 2000a). While water security often lays predominant focus on water supply (Grafton 2017; Jepson et al. 2017), the emerging domains of water scarcity have been recently considered, including: (1) human development, ecological sustainability, (2) geopolitics and international relations, and (3) vulnerability and risks (Jepson 2014). Where the term ‘security’ is invoked, a key question remains: ‘Security for whom?’—raising the need to understand who defines water security, how policies, discourses and practices are built around it, and how the costs and benefits of such interventions are distributed. By expanding these mixed conceptualisations in the Mekong Delta context, our study examines how water security is framed in state-society relations characterised by contested dynamics between (state-led) water management policies and (farmer-led) livelihood practices.

Previous studies indicate that water security is intertwined with efforts of agrarian societies, especially in the Global South, to ensure food security, food nutrition, and human well-being (Sinyolo et al. 2014; Bacon et al. 2021; Young et al. 2021). The concept also allies with strategies to build livelihood resilience in the context of water scarcity (Ranganathan et al. 2018; Aguilar et al. 2022). Interventions made towards achieving water security cannot solely resort to conventional water management approaches (Pahl-Wostl 2015). Rather, it entails the proactive realisation of risks and actions that water insecurity still exists and can be managed (Lankford et al. 2013). Achieving water security, therefore, points to the capacity of a society to manage water resources (Julio et al. 2021), where solutions need to be built on environmental and social lessons of the past (Grey and Sadoff 2007), as well as the capacity to deal with present and future water conditions. Considering these theoretical underpinnings in the contemporary water management challenges in the VMD, the study highlights the central role of the adaptive management approach that would help navigate learning pathways towards achieving water security in the long term.

Water security has emerged as a critical issue in transboundary river basins where vulnerability and complexity are high (Albrecht and Gerlak 2022; Yong 2022). In this context, transboundary water security challenges are conceptualised as water availability or scarcity, depending on how water is used at other geographical scales (Albrecht and Gerlak 2022). In most cases, the challenges are associated with ways riparian countries harness water for their own purposes, for example, hydropower development for economic growth (Moran et al. 2018), causing tensions between upstream and downstream users of water, and between local, national, and regional priorities (Lebel et al. 2005). Water security is therefore concerned with of how transboundary water flows could be maintained to secure the fair distribution and utilisation of water across geographical river boundaries (Munia et al. 2016). This is particularly evident in the Mekong region, where climate change and upstream hydropower dams exert substantial influence on the distribution of water resources and associated livelihood systems in downstream areas, including the VMD (Hoang et al. 2019).

Securing water for water-dependent livelihoods in the VMD, therefore, becomes a central issue to decision makers across water sectors and agrarian societies themselves. While leveraging efforts in water diplomacy, dialogues, and legal arrangements to tackle the transboundary water challenges at the regional and national scale are crucial (Mirumachi 2020; Yong 2022), it is equally important to investigate how the adaptive water management approach can contribute to the climate-resilient and sustainable development agenda at the delta scale (GoV 2017).^1^ The evidence in the case studies will illuminate how these endeavours have been invested in addressing the new water challenges in the delta.

### Adaptive management

Adaptive management has gained traction as an instrumental approach to environmental management, especially in the domains of water and flood management (Pahl-Wostl et al. 2007). It is introduced to address the perceived shortcomings of the IWRM^2^ (Integrated Water Resources Management) approach (Biswas 2008), whereby the overly broad and problematic discourses and practices (integrating multiple sources of data and knowledge into decisions) associated with IWRM have posed a hindrance to addressing needs for “pragmatic politics and solutions to water challenges” (Giordano and Shah 2014, p. 365). Instead, addressing these complex issues of water management would be based on ‘learning by experimenting’ (Varady et al. 2016). From the adaptive management perspective, this form of intervention, which involves a continuous cycle of planning, doing, monitoring, and evaluating (Schoeman et al. 2014), can assist in tackling such water challenges.

Learning is at the core of adaptive management. Through learning processes, valuable lessons learned from past management policies and actions provide effective responses to current environmental circumstances. Adaptive management encompasses the process of knowledge accumulation based on the structured cycle of conceptualising, monitoring, reflecting, learning, and adapting (Fabricius and Cundill 2014). At the institutional level, the concept demonstrates how learning outcomes among relevant stakeholders, including decision-makers and experts, when addressing complex, uncertain, and changing environments, can be obtained and applied in decision-making processes (Varady et al. 2016; Williams and Brown 2018). Given recurring water scarcity events in the VMD, it becomes imperative to understand how adaptive management facilitates learning among social actors, integrating the pools of knowledge into actions to deal with the situation. This points to the significance of hybrid water governance arrangements (Miller et al. 2019) that could be deployed to engage multiple stakeholders in decision-making processes to yield desirable environmental outcomes.

Adaptive management addresses drawbacks from the ‘command-and-control’ approach, assuming natural systems as predictable and stationary (Milly et al. 2008). This model aims to maximise resource exploitation, based on the centralised and expert-led problem-solving approach, and technical engineering solutions (Schoeman et al. 2014; Pahl-Wostl 2020). Under the authoritarian governance system in Vietnam, water management is predominantly directed by the ‘command-and-control’ approach (Käkönen 2008; Bruun and Rubin 2023). In the VMD, albeit having relatively efficient impacts on water problems through iterative ‘technological fixes’ (Pahl-Wostl et al. 2007), this approach still falls short of capacity to tackle the emerging water challenges under the combined impacts of climate and water infrastructure development at the regional and local scales (Tran and Tortajada 2022). Identifying these institutional and technical drawbacks allows reflective learning and the rectification of contemporary water management approaches to better respond to the Mekong’s changing waterscapes.

## Materials and methods

### Selection of the study areas

**Fig. 1:**
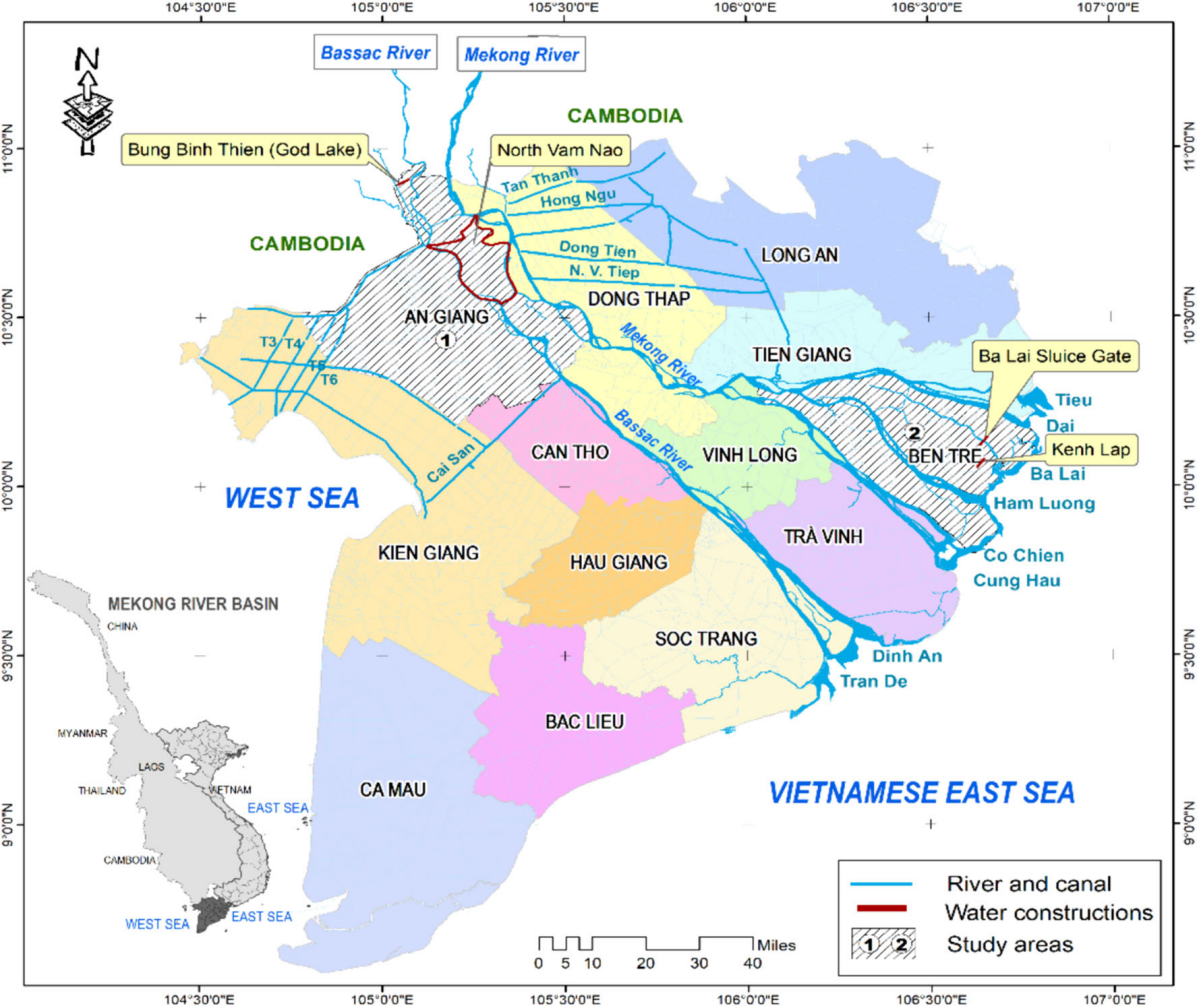
The study sites: (1) An Giang and (2) Ben Tre and water infrastructure in the VMD

Two provinces representing two ecologically distinct areas in the VMD were selected for the study. They include: An Giang in the floodplains (upper delta) and Ben Tre in the coastal zone (lower delta) (Fig. 1). The provinces have been exposed to recurring water scarcity events under the compounding effects of upstream hydropower operations, climate change, and local water infrastructure systems (dykes and canals) (Tran and Cook 2024). Given their enriched accounts on the policy shift of water management undertaken over the past few decades, the provinces provide the exemplary cases for the justification on how adaptive management has become an essential approach to address water scarcity and achieve water security in the long term.

An Giang Province is situated in the upper part of the VMD. Its geographical location (bordering Cambodia to the North) makes it heavily influenced by the hydrological regime of the Mekong River (see Fig. 1). An Giang is traditionally prone to high floods (≥ 450 cm), which, despite causing deep inundation on a large scale, provide favourable environments for agricultural and aquacultural production (Le et al. 2007). The combined effects of climate change (e.g. droughts), upstream hydropower dams, and the large-scale investment in local water infrastructure (i.e. dyke and drainage canal systems) have recurrently altered the seasonal water flows (Hoang et al. 2019), causing hardships for most rural inhabitants’ livelihoods.

Ben Tre is in the lower part of the VMD. Its proximity to the East Sea makes the province highly susceptible to saltwater intrusion (Tran et al. 2021). Local livelihoods are shaped by the distinct distribution of water systems, where freshwater occupies a large portion of the upper part and the brackish and saline water in the lower part of the province. Farming systems in the freshwater zones mainly include rice, bananas, and citrus fruits, while in the brackish and saline water zones intensive/extensive shrimp farming systems dominate (Table 1). Ben Tre has experienced extreme water scarcity in recent years (Tran and Cook 2024), posing multiple challenges to rural inhabitants whose livelihoods depend on water-based resources.

**Table 1:** Descriptive summary of the study areas.* Sources*: General Statistics Office (2022); Tran and Cook (2024); Tran et al. (2021); Interviews with key informants

Categories	The selected study areas	
	An Giang Province	Ben Tre Province
Geographical location	Upper delta (floodplains)	Lower delta (coastal plains)
Total land area (km^2^)	3537	2380
Population (people)	1 909 500	1 295 700
Density (person/km^2^)	540	544
Hydrological characteristics	Directly influenced by the seasonal floodwater regime from the Mekong River and the floodplain-based ecosystems	Directly influenced by the coastal ecosystems and the semi-diurnal tidal regime of the East Sea and indirectly influenced by upstream flow regulations
Proximate causes of water scarcity	El Niño effects and the upstream hydropower dams leading to reduced flows of flood	El Niño effects combined with the high magnitude and intensity of saltwater intrusion
Key water management systems	Large-scale flood control systems (e.g. North Vam Nao scheme), ring and embankments (high/low dykes)	Riverine salinity control and freshwater retaining systems (e.g. Ba Lai sluice), infield freshwater reservoir (Kenh Lap)
Primary livelihood activities	Dominant forms of agricultural and aquacultural production (e.g. rice, cash crops, fruits, catfish), flood-based livelihood activities (aquatic vegetables and species)	Rice, bananas, and fruits (freshwater zones), upland crops, brackish and saltwater farming systems (intensive/extensive shrimp), and integrated farming systems (shrimp-rice)

### Data collection and analysis

Qualitative research methods were employed to examine how the paradigm shift in water management paves ways for addressing water scarcity and achieving water security in the study areas. Data collection involves the undertaking of an extensive fieldwork together with three plenary stakeholder consultations, involving the participation of provincial government officials (e.g. Department of Agriculture and Rural Development), environmental and agricultural experts, and local farmer representatives (Table 2). We involved 10–15 participants in each session. A total of twenty-five interviews were conducted with key informants, including government officials working across administrative levels together with senior farmers who had profound experiences in dealing with water scarcity. The data collection occurred at multiple points in time: April and June 2019, April 2021, May and December 2022, and March and April 2024, and was undertaken both in-person and over Zoom. We also approached environmental experts for multiple interviews to obtain their perceptions of changing water environments in the VMD and the case study areas. The aggregation of these empirical data allowed the authors to thoroughly examine how climate change and infrastructure development influence water regimes at the transboundary and in-situ scales over time, and how they have enabled structural and institutional changes in water management.

**Table 2:** Summary of the data collection in the study areas

Methods	Research institutions	Government agencies and farmers in the study areas			
		An Giang Province		Ben Tre Province	
	Key informants involved	Key informants involved	Information collected	Stakeholders involved	Information collected
In-depth interviews (in person and online)	Agricultural and hydrological experts from local and regional research institutions	Department of Agriculture and Rural Development Department of Natural Resources and Environment An Phu Office of Agriculture and Rural Development An Phu Office of Environment and Natural Resources Rice and fish farmers in An Phu Phu Tan Office of Agriculture and Rural Development Phu Tan Office of Environment and Natural Resources Rice and fish farmers	Change in water systems Drivers of flood fluctuations and impacts on local livelihoods Responses to emerging water challenges Change in water management at institutional and household levels Response to altering water systems (water disruptions) facing farmers’ livelihoods	Department of Agriculture and Rural Development Department of Natural Resources and Environment Binh Dai Office of Agriculture and Rural Development Binh Dai Office of Natural Resources and Environment Farmers in freshwater and saltwater zones	The progress and impacts of saltwater intrusion and water scarcity in the province Water management infrastructure (Ba Lai sluice system, Kenh Lap reservoir) in responding to saltwater intrusion and supply of freshwater (addressing water scarcity) Adaptive livelihoods in times of change (saltwater intrusion and water scarcity)
Stakeholder workshops		Leaders of provincial government agencies, agricultural and environmental experts, and farmer representatives	Emerging water challenges facing the province Water scarcity and impacts on agricultural and aquacultural production Policy recommendations for water management (addressing water scarcity)	Government officials from the Department of Agriculture and Rural Development	Processes of saltwater intrusion, impacts, and solutions to water management Policy recommendations for water management (addressing saltwater intrusion and ensuring water supply)

Apart from the primary data obtained from the stakeholder consultations and interviews, we also conducted desk studies on a wide range of reference materials, including academic articles, books, government policy documents, conference recordings, and scientific reports. These mixed sources of references provide the contextual knowledge of the study, allowing the authors to gain better insights into water issues, especially on an ongoing shift in water management and adaptive pathways towards achieving water security in the VMD.

We used the thematic strategy for analysing the data. Specifically, we conducted an integrated deductive and inductive thematic analysis guided by Fereday and Muir-Cochrane (2006) (see Supplementary material). This technique allowed us to profoundly capture the meaning of the data (Lochmiller 2021), whereby gaining better insight into how water scarcity has become an unprecedented environmental phenomenon in the VMD, and how adaptive management offers a pathway towards addressing the problem. In this study, the use of document analysis helps complement the primary data, making the analysis more inclusive and robust.

## Results

### The Mekong Delta’s water scarcity

The delta’s waterscapes have undergone substantial transformation. Most notably, water scarcity has become increasingly prevalent across the delta. At the consultation with stakeholders in An Giang Province, for instance, a senior official from Department of Agriculture and Rural Development (DARD) highlighted that “Canal systems in the province do not provide sufficient water for irrigation. Farmers have to pump water into their fields, resulting in increased production costs” (Stakeholder consultation, May 2022). Our data indicated that three factors are perceived to contribute to water scarcity facing the delta, including El Niño effects of climate change, the water storage of upstream hydropower dams, and the Vietnamese government’s long-lasting pursuit of intensive agriculture-oriented development policies. These reasons were articulated by a Mekong expert in the following quote:

Water scarcity in the VMD is largely attributed to compounding effects of climate change, upstream hydropower dams, and delta-wide efforts in building water infrastructure systems for agricultural production (Interview, November 2020).

Our analysis suggests a paradoxical legacy in the state-led water management policies. Attempts to convert the delta into a highly productive agricultural frontier demanded that the surplus floodwater flowing into the VMD be forcibly expulsed from the floodplains. This involved the building of lateral drainage canals that connected the Long Xuyen Quadrangle to the West Sea (see Fig. 1). These systems aimed to achieve four main goals: (1) drain out surplus floodwater from the delta, (2) reduce the level of acid sulphate in soil, (3) provide flood-free conditions for the cultivation of multiple rice cropping systems, and (4) reduce flood risks for flood-prone communities. The systems are believed to have a substantial influence on water regimes in the delta. A hydrological expert noted that:

Lateral canals to the West Sea have profound implications for the water conditions we are currently facing. These systems may contribute to water scarcity and the encroachment of saltwater during the dry season (Interview, June 2022).

**Fig. 2:**
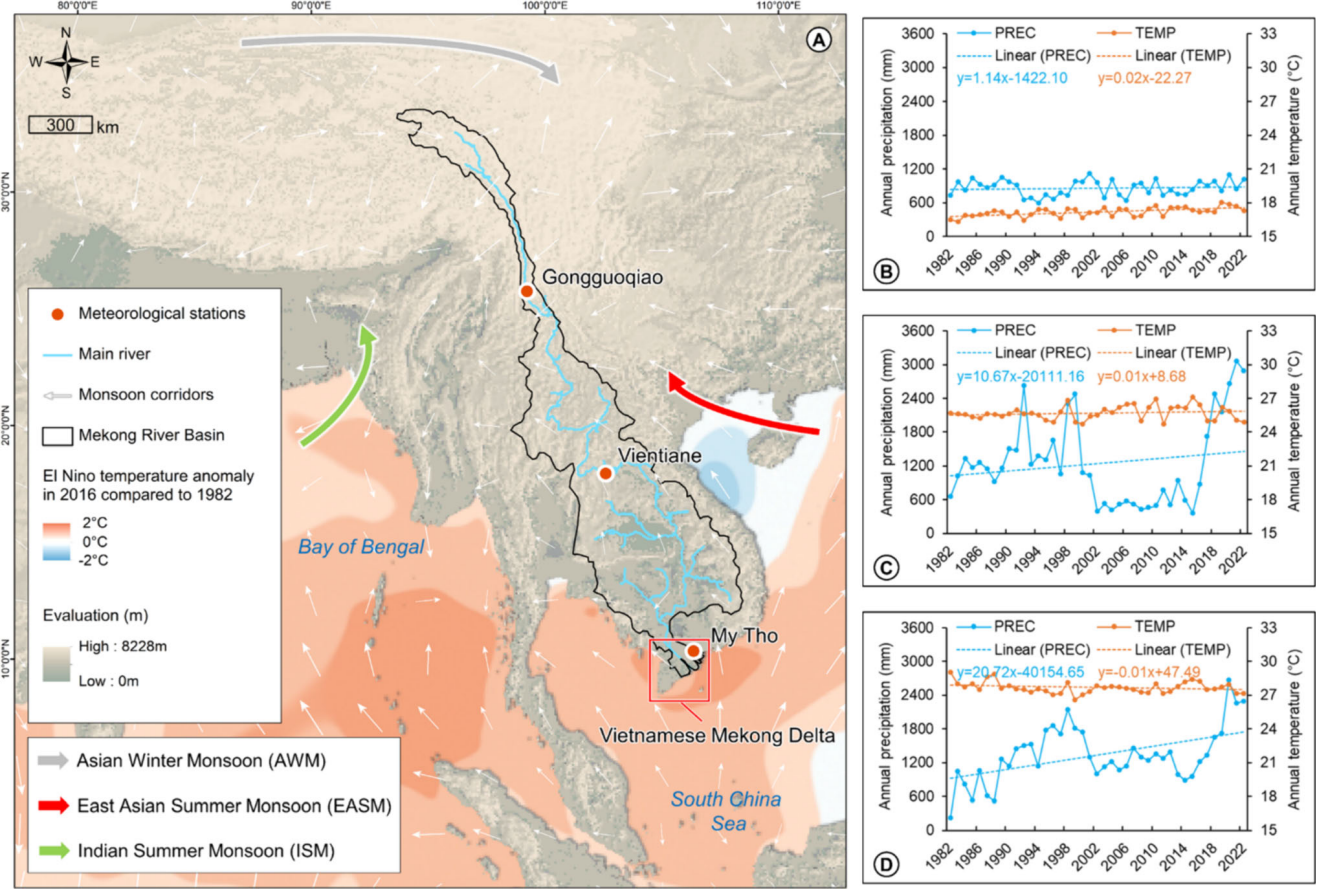
The Mekong region and the VMD under the extreme El Niño events in 2016 compared to 1982 (**A**) along with variations in precipitation and temperature at three meteorological stations: Gongguoqiao (China) (**B**), Vientiane (Laos) (**C**), and My Tho (Vietnam) (**D**) over the period 1982–2022. Sources: Scientific Visualisation Studio and Power Access Viewer (NASA)

Changing temperature and precipitation from El Niño effects significantly impacts water supply in the Mekong region (Fig. 2), contributing water scarcity in the VMD. We observed a temperature increase of 2 °C during the El Niño event in 2016 compared to 1982 (Fig. 2A), evidenced by the increasing trends (slopes > 0) in the yearly temperature recorded at the Gongguoqiao and Vientiane meteorological stations between 1982 and 2022 (Fig. 2B–D). This period also witnessed an increasing trend in the yearly precipitation at all three stations. While much of the Mekong water is trapped in upstream hydropower reservoirs, the reduced rainfall under the El Niño effects (as seen in the 2002–2016 period described by Fig. 2B and C) does not secure the water supply for the VMD. These regional processes, compounded with the in-situ impacts of the water expulsion policies, have aggravated water scarcity in the delta. These present challenges to sustaining its productive agro-ecosystems which serve as a pivotal means of livelihoods for the majority of rural inhabitants.

On the other side of the Mekong floodplains, Cambodia is expediting the construction of a 180-km Funan-Techo canal that links Phnom Penh and the Cambodian coast (the Gulf of Thailand) in support of its inland waterway transports. From the transboundary perspective, this work is predicted to present more water challenges to the VMD. Although Cambodia claims the canal will have no significant impacts on the Mekong River’s hydrological flows (CNMC 2023), the Vietnamese government still holds their critical concerns over its effects on the delta’s environment and water regimes (e.g. aggravating water scarcity) (Sok 2024).

In the coastal zones, the national government’s exercise of the ‘freshening the coastal zones’ policy has changed the nature of the coastal waterscapes (Tran et al. 2022b). While these structural systems sought to accommodate local needs (e.g. water supply and reduction of saltwater intrusion), they come to have disturbance on local farming systems. The anecdotal evidence gathered from the first author’s conversation with a local farmer indicated that farmers in the freshwater zones (the upper stretch of the Ba Lai River) have experienced the big loss of coconut harvests due to high exposure to sulphate and salinity-affected soil conditions. He noted that “Not only coconut trees, but a similar situation is also found with the citrus trees (e.g. oranges) that are adversely exposed to salinity. This leads to the mass chopping-down just after a few years of cultivation” (Interview, December 2022).

### ‘Quantity’-oriented water management approach

The water management approach through water retention obviously give more priority of water quantity over water quality. Our data suggested that in the face of harsh water scarcity, the governments have proactively implemented water retention policies to store as much water as possible. This is to secure sufficient water supply for agricultural production and household water consumption. A participant from the stakeholder consultation in An Giang noted that “The biggest challenge we are facing is water scarcity. We need to invest in water infrastructure systems to retain water” (Stakeholder consultation, May 2022).

Water retention depends on how various infrastructure systems could be used. This includes the adoption of grey and green infrastructure solutions (Tran and Cook 2024). In An Giang Province, water is retained in dike compartments (e.g. Bac Vam Nao scheme), canals, and natural wetlands. In Ben Tre Province, water is stored in reservoir schemes (e.g. Ba Lai and Kenh Lap) (see Fig. 1). The accelerating rate of saltwater intrusion in the province necessitates the storage of a large volume of freshwater in these systems to meet local needs. A local government official stressed how the Ba Lai scheme makes it possible:

The district has experienced the dearth of freshwater during the dry season. It is fortunate to have the national government’s investment in the Ba Lai system that helps retain freshwater for agricultural production household consumption (Interview, April 2021).

### Learning through adaptive water management

**Fig. 3:**
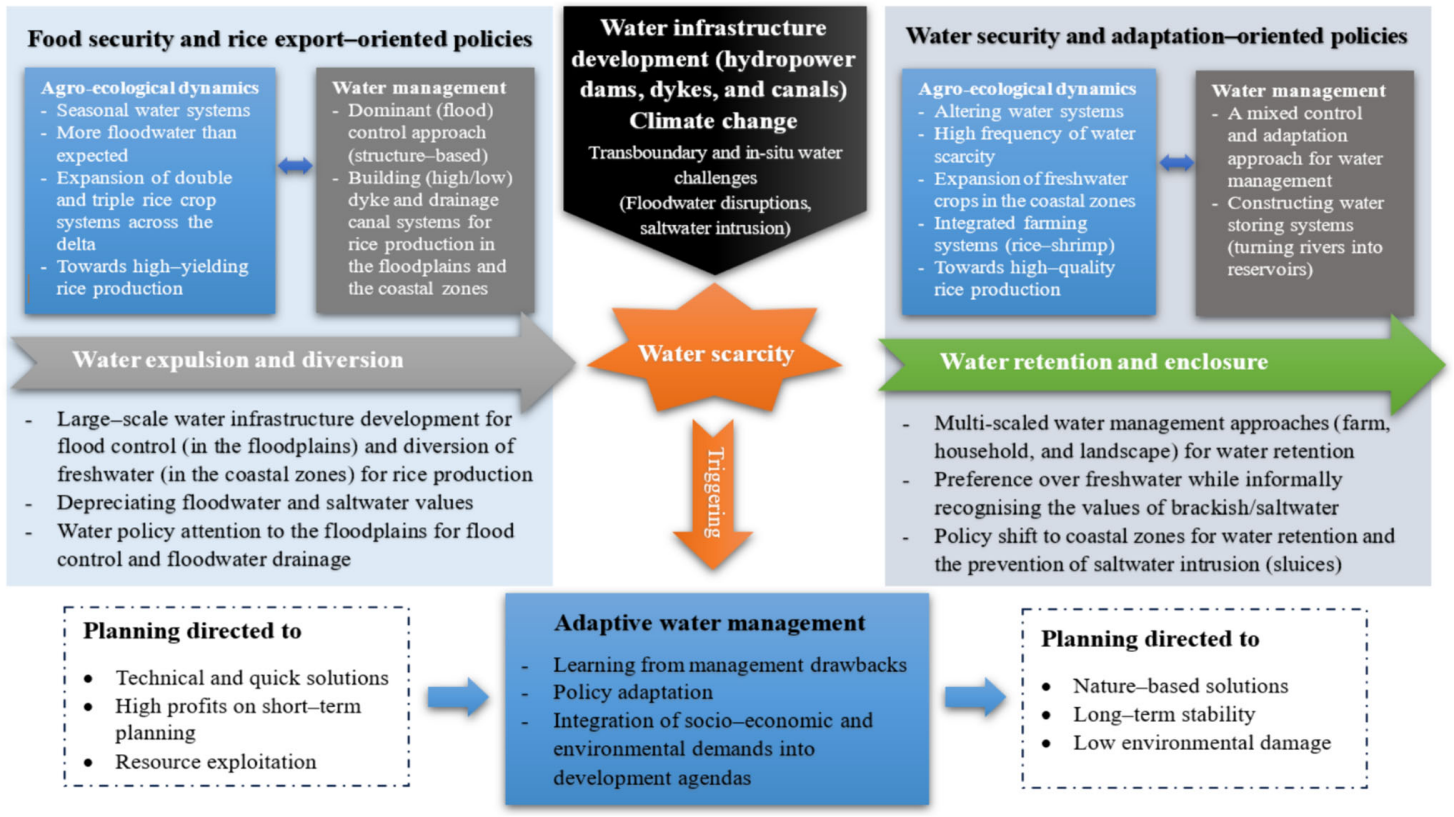
The evolving pathways of adaptive water management in addressing water scarcity and achieving water security in the study areas

Adaptive water management lends itself to continuous learning to address water challenges over time. In the VMD, the paradigm shift from water expulsion to water retention presents salient evidence of policy learning in water management (Fig. 3). This process is initially concerned with the state-led experimentation of new water management policies to attain their political goals (e.g. food security) and followed by the modification of those policies to accommodate new water conditions (e.g. water security) and socio-economic development agendas.

Learning draws on the predicaments perceived in the operation of water infrastructure systems. In the floodplains, the water expulsion policy did not envision how it contributes to saltwater intrusion and water scarcity at present. A senior government official involved in the water expulsion policy admitted that “I agree that the development of water infrastructure in the VMD, as seen in the water expulsion policy, has its periodic implication. It lacks a long-term vision to deal with water challenges as we are currently facing” (Interview, March 2024). In Ben Tre Province, the construction of the Ba Lai case study, the sealing-off of the river estuary, while enabling the prevention of saltwater intrusion and preservation of freshwater, triggered contestations between agrarian communities and the local government. This was evidenced by local farmers’ keen pursuit of traditional livelihoods (e.g. shrimp farming), which is not aligned with the government’s policy towards freshwater crop development in the freshwater zones.

Learning enables change in water management decisions. Until harsh water scarcity events had occurred, the provincial governments began to realise the significance of water retention (Table 3). A government official in An Giang noted that “some 3000 hectares of farmland in Tinh Bien District will be devoted to water retention following the cultivation of two rice crops” (Interview, May 2022). The consultation with An Giang government officials indicated that the provincial government has been pushing an agenda for building large-scale reservoirs for water retention. Obviously, grey infrastructure is adopted as a key solution (Tran and Cook 2024). However, there remains doubt about how this ad-hoc solution would be efficiently undertaken to achieve sustainable water security in the longer term. A hydrological expert noted:

I am concerned that the building of large-scale reservoir for water retention would be costly and exposed to high evaporation. Various water retention approaches, therefore, need to be considered (Interview, June 2022).

**Table 3:** Water management approaches and changes in decision-making in the study areas.* Sources*: An Giang People’s Committee (2013)^a^; Tran and Cook (2024)^b^; Tran et al. (2022b)^c^; Tran and Pittock (2024)^d^; Interviews with key respondents

Case studies	Water management approaches undertaken	Impacts	Learning for change in decision-making processes
In the floodplains (An Giang Province)	Water expulsion to drain out floodwater to the West Sea Building high dikes for intensive rice production	Drying out the delta leading to extreme water scarcity in the dry season Attributing to the emergence of saltwater intrusion Minimising the entry of floodwater into fields, causing the loss of soil fertility	Recognition of water values in the wake of water scarcity Deferral/termination of funding support to building high dikes^a^ Water retention^b^
In the coastal zones (Ben Tre Province)	Sealing off the riverine estuaries^3^ Converting the coastal zones (from brackish/saltwater to freshwater systems)	Changing the local ecosystems Engendering contestations between the local government and agrarian communities (e.g. shrimp farmers)^c^	Water retention^b^ Recognising the values of brackish/saltwater in supporting coastal livelihoods Relaxing the policy on freshwater-oriented crop production in the freshwater zones^d^

**Fig. 4:**
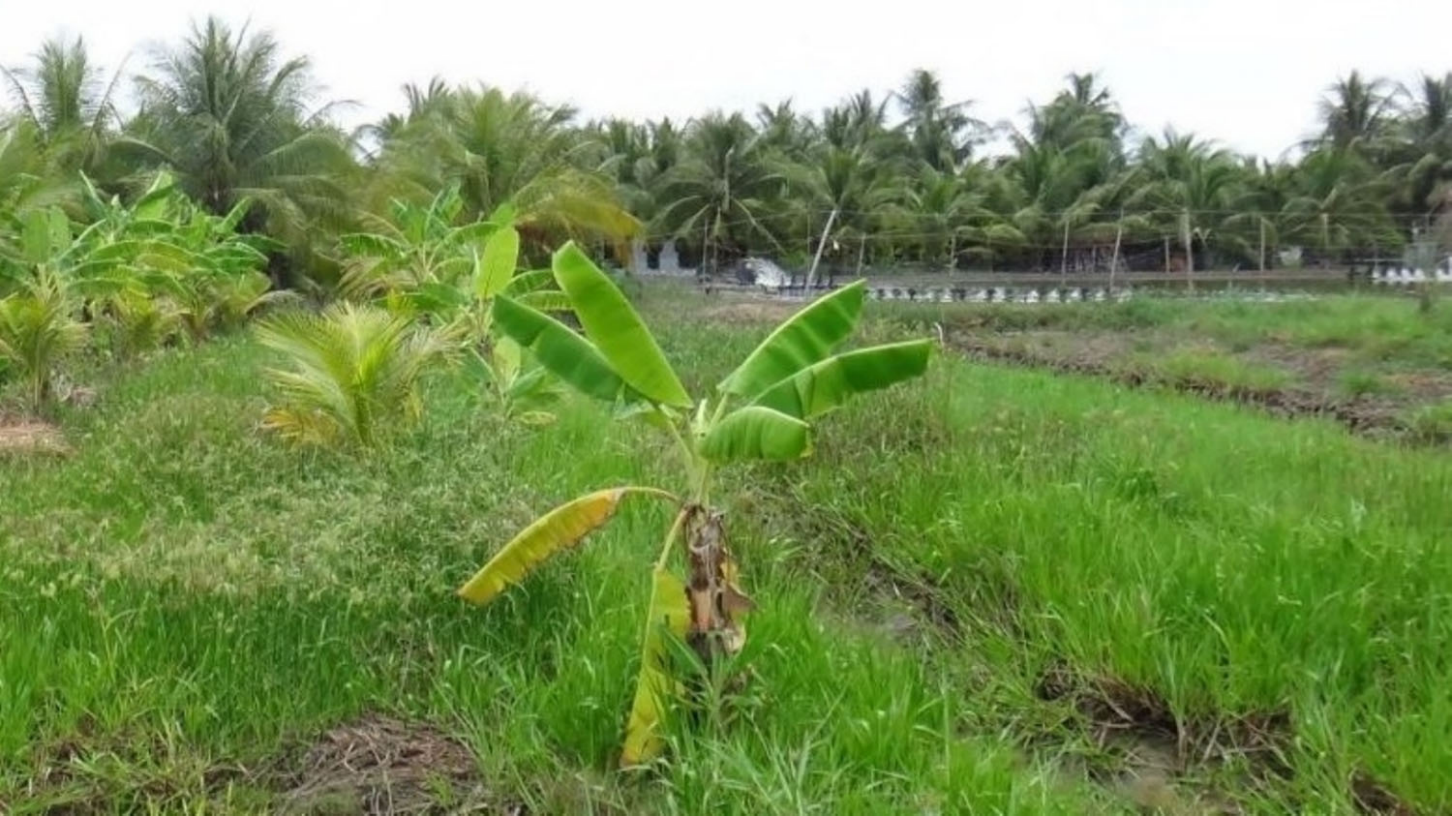
Shrimp farming in the freshwater zones of the Ba Lai case study. *Source*: The first author

Learning was evidenced by a change in the local government’s perception of water resources. While freshwater conventionally gains more priority, especially in agricultural production, brackish/saltwater resources are recognised as making valuable contribution to coastal zones’ economies. For the Ba Lai case study, brackish/saltwater, despite being strictly prohibited in the freshwater zones, holds paramount importance for shrimp farming. Given its undeniable profitability compared with other freshwater agro-products (e.g. rice), shrimp farming is persistently practised in the freshwater zones (Fig. 4) and was somehow ‘blindly accepted’ by the local government (Tran and Pittock 2024).

### Moving towards sustainable water security in the *delta*

The policy shift from prioritising food security and rice export demands to water security and adaptation-based sustainable development suggests a transformative pathway in achieving sustainable water management (see Fig. 3). From the policy perspective, this adaptive pathway suggests the nuanced significance of how water management is incorporated into local development policies to bring about desirable outcomes. It allows local governments to pursue their economic development agendas while seeing water security as key to warrant the efforts. A government official involved in the stakeholder consultation in An Giang highlighted:

Concerning the future of the VMD, we should not keep waiting for expected outcomes from negotiating with upstream Mekong countries, we need to proactively store water in the delta (Stakeholder consultation, May 2022).

Managing change towards adaptive water management needs to attend to solutions to water and livelihood politics. The Ba Lai case presents a dilemma in how water retention supported by the scheme disturbed coastal traditional livelihood systems. The priority of freshwater over brackish/saltwater policy did not accommodate demands from agrarian communities, who contended that shrimp farming is the most productive livelihood option *vis-à-vis* freshwater agro-commodities. Addressing these contestations will contribute to securing the equitable distribution of water resources for the delta’s coastal livelihoods while seeking solutions towards achieving water security in the long term.

## Discussion

### ‘Food-to-water’ security policies

National food security and rice export demanded by the national government dictated the expansive investment in infrastructural systems (canals, dikes) across the VMD to implement the ‘rice everywhere’ policy (Biggs et al. 2009) during the post-Renovation period. This comes with the emphasis on intensive agricultural production and approaches to control water regimes to make it possible. Expulsing excessive floodwater and converting the coastal zones into a new agricultural resource frontier by cultivating two or three rice crops per year was carried out across the delta. The adoption of this technocratic ideology creates an inevitable legacy for the rectification of water management policies to deal with prevailing water challenges (e.g. water scarcity) facing the delta.

Expanding water infrastructure for rice-based agricultural development in the delta represents a political mandate of the national government to modernise the rural economy and enhance the income of rural agrarian societies. Here, big water infrastructure could be an outcome and driver of modernisation (Birkenholtz 2023). As evidenced in the floodplains, the development of floodwater drainage systems contributes to water disruptions, while unexpectedly paving pathways for saltwater encroachment into inland zones. As such decisions were largely bound to short-term development planning (Tran 2020), they failed to take account of potential effects of climate change and upstream hydropower operations that have now plagued the delta.

Water disruptions of the Mekong River driven by upstream hydropower operations, El Niño effects, and the water expulsion policy have turned the VMD from a water-abundant into unprecedentedly water-scarce delta (Hecht et al. 2019; Tran and Tortajada 2022). This corresponds to Vörösmarty et al.’s (2010) perception that the widespread presence of engineering works would lead to the overuse and mismanagement of water resources. It also comes at odds with the ‘normative’ flood regime that major floods appeared every five years (Sneddon and Binh 2001). Water scarcity could be the consequence of the state’s water mismanagement and the short-term land use planning in rice-based agriculture development.

### Water scarcity as a leverage point for policy change

Water scarcity sets a leverage point for change in water management policies. First, the rectification of water management policies (water retention) means that there is a shift from the depreciation to appreciation of water values (see Fig. 3). Here, water retention suggests that the delta’s agrarian societies are making efforts to secure a year-round supply of freshwater (whether in the floodplains or the coastal zones) under the interlocking effects of climate-development dynamics (Tran and Cook 2024).

There exists institutional inertia in addressing the new water complexities. From our case studies, ‘fixing’ endeavours are not adequately grounded in the knowledge of climate and development dynamics at the regional and local scales. There was also a lack of environmental and geopolitical understandings of Mekong’s altering water regimes in relation to local water management policies in place, thus undermining institutional capacity to proactively diagnose and learn, and resolve the water problems.

However, failures to achieve sustainable water management, as Schoeman et al. (2014) put it, prompt new ways of perceiving and acting with water. In our case studies, this learning has leveraged efforts for water retention across scales (farm, household, and landscapes) and across ecological zones (floodplains and coastal zones) (Tran and Cook 2024). While these ‘revamping’ approaches help address the water problems (water scarcity) delta-wide, some doubts remain as to how they could be managed to deliver desirable outcomes.

Promoting the adaptive water management approach in the VMD urges the reframing of the existing water governance framework to better address the new water challenges. Here, two issues need to be taken into account. First, there should be the formal recognition that water retention—as proactively deployed across the case study areas (An Giang and Ben Tre) —would not be sufficient to tackle the acute effects of water scarcity and achieve water security in the long term. This sets a stage for leveraging collaborative water management among jurisdictions and ecological zones (e.g. floodplains and coastal zones) in the quest for climate resilience and sustainable development in the VMD mandated by Resolution 120. A successful case for this effort is the formulation of a collaborative water management agreement between An Giang and Kien Giang in 2013 (Tran et al. 2019). These arrangements allow farmers in Kien Giang to organise their crop cultivation to avoid possible crop damage and other environmental consequences from seasonal floodwater drainage from their neighbouring province. Second, water-livelihood relations should be put at the centre of adaptive water management policies. Given the lessons learned from the Ba Lai case study, the household economy based on the utilisation of brackish/saltwater resources needs to be integrated in local development agendas, where environmental and economic costs and contestations between the local government and farmers could be minimised and avoided. It is expected that resolving these relational problems will help enhance capacities for achieving water security and sustainable livelihoods in the delta.

## Conclusions

The paper presents how adaptive water management has become an essential approach towards achieving water security in the VMD. The case studies demonstrate how infrastructure-based water management practices have contributed to water challenges, such as water scarcity and saltwater intrusion. From the perspective of adaptive water management, the paper presents an ongoing shift in water management from changing the focus from food security to water security in tackling the emerging climate-development complexities.

The paper contributes to advancing the theoretical and empirical understandings of how water security could be achieved in the VMD. We argue that this effort should go beyond simplistic water retention solutions (i.e. sealing off rivers and building water-storing infrastructure). Water quality, a key component of water security, should be integrated in water management policies, which often gives more priority to water quantity. While freshwater gains a legitimate interest by the local government in enabling pro-agriculture development policies in coastal zones, the role of brackish/saltwater resources should be equally recognised in bolstering the local economy. These institutional realignments would also help avoid water-livelihood contestations.

Achieving water security in the VMD needs to leverage efforts in water policy consultations and diplomacy that go beyond its geographical scale. It is essential for the delta’s governments to engage in dialogues with a multitude of stakeholders, including NGOs (Non-Governmental Organisations), the Vietnam National Mekong Committee, the Mekong member countries, as well as international agencies to articulate their concerns over the in-situ and transboundary water challenges facing the VMD and coordinate efforts to address the problems. Future research work needs to disentangle this space by investigating how these forms of engagements enable change in water politics and policy actions in the delta.

## Supplementary Information

Below is the link to the electronic supplementary material.

Supplementary file1 (PDF 483 kb)

## Footnotes

1 This flagship document (known as Resolution 120) was promulgated in 2017, aimed to guide the national government’s directions on implementing climate–resilient and sustainable development in the VMD. It entails an overarching development agenda which includes long–term visions, objectives, solutions as well as concrete tasks to improve economic development, while prioritising adaptation as a pivotal approach to deal with climate change.

2 This model is defined as a “process which promotes the coordinated development and management of water, land and related resources, in order to maximise the resultant economic and social welfare in an equitable manner without compromising the sustainability of vital ecosystems” (Global Water Partnership 2000b, p. 22).
