# Supplementary material for: Evolving pathways towards water security in the Vietnamese Mekong Delta: An adaptive management perspective
Source: Ambio. 2024 Jun 29;54(3):460–74. doi: 10.1007/s13280-024-02045-0 (PMC11780020; doi:10.1007/s13280-024-02045-0)
Supplement: Supplementary file 1 — Supplementary file1 (PDF 483 kb) [file 13280_2024_2045_MOESM1_ESM.pdf]

Supplementary Information

Evolving pathways towards water security in the Vietnamese Mekong Delta: An adaptive management perspective

Thong Anh Tran [a, b, *], Dung Duc Tran [c, d], Oc Van Vo [b], Van Huynh Thanh Pham [e], Hieu Van Tran [e], Ming Li Yong [f], Phu Viet Le [g], Phu Thanh Dang [b]

[a] Fenner School of Environment and Society, College of Science, The Australian National University, Canberra, Australia

[b] Climate Change Institute, An Giang University, Vietnam National University Ho Chi Minh City (VNU-HCM), Long Xuyen City, An Giang Province, Vietnam

[c] National Institute of Education, Earth Observatory of Singapore and Asian School of the Environment, Nanyang Technological University, Singapore

[d] Centre of Water Management and Climate Change, Institute for Environment and Resources, Vietnam National University Ho Chi Minh City (VNU-HCM), Ho Chi Minh City, Viet Nam

[e] Faculty of Agriculture and Natural Resources, An Giang University, Vietnam National University Ho Chi Minh City (VNU-HCM), Long Xuyen City, An Giang Province, Vietnam

[f] Research Program, East-West Centre, Honolulu, Hawai‘i, United States

[g] Fulbright School of Public Policy and Management, Fulbright University Vietnam, Ho Chi Minh City, Vietnam

[*] Corresponding author. Tel: (+61) 0431-629-270. Email address: thong.tran@anu.edu.au (T.A. Tran).

Supplementary Material

The process of conducting the integrated deductive and inductive thematic analysis based on Fereday and Muir-Cochrane's (2006) approach.

<table>
<tr><td>Deductive thematic analysis
(Theory–driven coding)</td><td>Inductive thematic analysis
(Data–driven coding)</td></tr>
<tr><td>- A template (codebook) was developed a priori based on the research questions sought to answer and the conceptual framework informed by the extensive reviews of relevant literature (examining concepts and ways they are linked to water conditions, water–based livelihoods, and water management approaches in various contexts (countries in the global South, transboundary river basins), and the VMD).
- Key concepts formed the template included: "water security", "adaptive management", "water management", "water–based livelihoods", "water expulsion," and "water retention."</td><td>- Preparation of (primary) qualitative data, which include:
▪ In–depth interviews with government officials working across administrative levels (provincial, district, and communal), and farmers directly affected by water scarcity
▪ Stakeholder consultations with provincial government officials, environmental and agricultural experts, and farmer representatives</td></tr>
<tr><td colspan="2">Summarising data and identifying initial themes</td></tr>
<tr><td>- Codes were developed from the template (codebook) and assigned to the text based on transcribed documents (e.g. in–depth interviews and stakeholder consultations) that have been imported into the NVivo project.</td><td>- Inductive codes developed from the transcribed documents introduced new themes. These emerging themes were located separately from predetermined nodes (free nodes) or expanded the pre–existing codes built from the template (tree nodes).</td></tr>
<tr><td colspan="2">Connecting the developed codes and identifying shared themes based on the research questions and the conceptual framework.
Themes were clustered and assigned phrases to describe the meaning that underpins the theme.
- Development of the analytical framework for the study based on the concepts in the template and themes emerged from the analysis (see Fig. 3). The analytical framework serves as a roadmap that guides the development of the paper.</td></tr>
</table>
